# Cut-Off Value of Total Adiponectin for Managing Risk of Developing Metabolic Syndrome in Male Japanese Workers

**DOI:** 10.1371/journal.pone.0118373

**Published:** 2015-02-23

**Authors:** Akiko Hata, Koji Yonemoto, Yosuke Shikama, Nanako Aki, Chisato Kosugi, Ayako Tamura, Takako Ichihara, Takako Minagawa, Yumi Kuwamura, Masashi Miyoshi, Takayuki Nakao, Makoto Funaki

**Affiliations:** 1 Clinical Research Center for Diabetes, Tokushima University Hospital, Tokushima, Japan; 2 Biostatistics Center, Kurume University, Fukuoka, Japan; 3 Department of Medicine and Bioregulatory Sciences, The University of Tokushima Graduate School of Medical Sciences, Tokushima, Japan; 4 Department of Nursing Science, Institute of Health Biosciences, The University of Tokushima Graduate School, Tokushima, Japan; 5 Clinical laboratory, Tokushima University Hospital, Tokushima, Japan; Hamamatsu University School of Medicine, JAPAN

## Abstract

**Aim:**

To determine the optimal cut-off value of serum total adiponectin for managing the risk of developing metabolic syndrome (MetS) in male Japanese workers.

**Methods:**

A total of 365 subjects without MetS aged 20–60 years were followed up prospectively for a mean of 3.1 years. The accelerated failure-time model was used to estimate time ratio (TR) and cut-off value for developing MetS.

**Results:**

During follow-up, 45 subjects developed MetS. Age-adjusted TR significantly declined with decreasing total adiponectin level (≤ 4.9, 5.0–6.6, 6.7–8.8 and ≥ 8.9 μg/ml, P for trend = 0.003). In multivariate analyses, TR of MetS was 0.12 (95% CI 0.02–0.78; P = 0.03) in subjects with total adiponectin level of 5.0–6.6 μg/ml, and 0.15 (95% CI 0.02–0.97; P = 0.047) in subjects with total adiponectin level ≤ 4.9 μg/ml compared with those with total adiponectin level ≥ 8.9 μg/ml. The accelerated failure-time model showed that the optimal cut-off value of total adiponectin for managing the risk of developing MetS was 6.2 μg/ml. In the multivariate-adjusted model, the mean time to the development of MetS was 78% shorter for total adiponectin level ≤ 6.2 μg/ml compared with > 6.2 μg/ml (TR 0.22, 95% CI: 0.08–0.64, P = 0.005).

**Conclusion:**

Our findings suggest that the cut-off value for managing the risk of developing MetS is 6.2 μg/ml in male Japanese workers. Subjects with total adiponectin level ≤ 6.2 μg/ml developed MetS more rapidly than did those with total adiponectin level > 6.2 μg/ml.

## Introduction

In recent decades, the metabolic syndrome (MetS) has received much attention, and many aspects of MetS, including its prevalence, incidence, and risk of leading to the development other conditions such as type 2 diabetes [1], cardiovascular disease [2] and stroke [3], have been widely reported. Moreover, adipocytokine secretion from adipose tissue, especially visceral fat, has been suggested to contribute to the development of MetS. Among adipocytokines, adiponectin has been reported to play a protective role in the development of MetS [4].

Adiponectin is secreted mainly by adipose tissue, and a high level is present in the bloodstream [5]. Adiponectin circulates in multimers, i.e., as high-molecular-weight (HMW), medium-molecular-weight, and low-molecular-weight adiponectin complexes [6]. Clinical and epidemiological studies have shown an inverse relationship between adiponectin and MetS and components of MetS [7–9]. Thus, serum adiponectin level has been expected to serve as a valuable biomarker to predict the development of MetS. However, cut-off values of total adiponectin, as well as other forms of adiponectin [10] to discriminate MetS have been mainly evaluated based on cross-sectional studies, so that the optimal cut-off value of total adiponectin for managing the risk of developing MetS remains a matter of debate.

The aim of the present study was to propose the optimal cut-off value for managing the risk of developing MetS based on data from a prospective cohort study of male Japanese workers, by using the accelerated failure-time (AFT) model, which is more appropriate than the Cox proportional hazards model [11].

## Materials and Methods

### Study participants and design

This is a prospective occupational-based study that has been underway since 2008 in Tokushima Prefecture, which is located in Shikoku Island of Japan. We recruited workers, aged 20 to 60 years. Briefly, 821 workers aged 20 to 60 years underwent a screening survey for the present study. In this study, we focused on male subjects only, so 550 male subjects were included in this study. After exclusion of 13 subjects who had already eaten breakfast, 69 subjects with MetS, and 1 subject without a blood sample, the remaining 467 subjects without MetS were enrolled in the baseline examination.

The baseline subjects were followed-up prospectively from 2008 to 2012 by repeated annual health examinations. Of the baseline subjects, 365 who underwent at least one re-examination were selected for the present study (follow-up rate, 78.2%). During the follow-up, 45 developed MetS.

### Definition of metabolic syndrome

MetS was defined by the criteria of the joint interim statement of the International Diabetes Federation Task Force on Epidemiology and Prevention, the National Heart, Lung, and Blood Institute, the American Heart Association, the World Heart Federation, the International Atherosclerosis Society, and the International Association for the Study of Obesity in 2009 [12]. Accordingly, MetS was defined as three of the following five criteria: (1) increased waist circumference: waist circumference ≥ 90 cm in men, (2) elevated triglycerides: serum triglycerides ≥ 1.7 mmol/l or use of drug treatment for elevated triglycerides, (3) reduced HDL-C: serum HDL-C < 1.0 mmol/l or use of drug treatment for reduced HDL-C, (4) elevated blood pressure: blood pressure ≥ 130/85 mmHg or use of antihypertensive medication, and (5) elevated fasting glucose: fasting plasma glucose ≥ 100 mg/dl or drug treatment for elevated glucose.

### Clinical evaluation and laboratory measurements

Fasting venous blood samples after an overnight fast of at least 10 hours were collected from each subject at the baseline and follow-up examinations. Plasma glucose level was determined using a commercially available glucose oxidase-peroxidase method (ADAMS glucose GA-1170, ARKRAY, Inc., Kyoto, Japan) [13]. Serum high-sensitivity C-reactive protein (hs-CRP) concentration was determined using a commercially available latex turbidimetric immunoassay (LT CRP-HS II, Wako Pure Chemical Industries, Ltd., Osaka, Japan) [14]. HDL-C concentration was determined using a commercially available direct method (QUALIGENT HDL, Sekisui Medical Co., LTD., Tokyo, Japan) [15]. Triglyceride concentration was determined using a commercially available enzymatic method (QUALIGENT TG, Sekisui Medical Co., Ltd., Tokyo, Japan) [16]. Sitting blood pressure was obtained in each arm, and the average value was used in the analyses. Body height and weight were measured in light clothing without shoes, and BMI was calculated.

Collected serum specimens were stored at -80°C until measurement of total adiponectin concentration, which was within a month. Serum adiponectin concentration was measured by latex immunity nephelometry (Otsuka Pharmaceutical Co., Tokushima, Japan).

Each subject completed a self-administered questionnaire covering medical history, anti-diabetic, anti-hyperlipidemic, and anti-hypertensive treatment, alcohol intake, and smoking habit at the screening. Alcohol intake and smoking habit were classified as either current or not current. At baseline examination, a physical activity survey was conducted using an International Physical Activity Questionnaire [17]. Subjects engaging in physical activity at least once a week during their leisure time were included in the regular-exercise group.

### Statistical analysis

Total adiponectin level was divided into four categories based on the quartile distribution: ≤ 4.9, 5.0–6.6, 6.7–8.8 and ≥ 8.9 μg/ml. Age-adjusted mean values of possible risk factors were calculated by analysis of covariance, and their trends across the quartiles of total adiponectin were tested by multiple regression analyses. Frequencies of risk factors were adjusted for age by a direct method, and their trends were examined using logistic regression models. AFT models were used to investigate the association between total adiponectin and the development of MetS, which put the emphasis on the time to an event [11]. The exponential, Weibull, and gamma distributions were chosen as candidates for the survival distribution for an AFT model [11, 18], and the one with the minimum Akaike information criterion (AIC) value was chosen as the survival distribution. Multivariate adjusted time ratio for the development of MetS was estimated by incorporating age, BMI, smoking habit, alcohol intake, and regular exercise into multivariate AFT models. Next, the optimal cut-off value of total adiponectin was explored using a multivariate AFT model with a binary variable that indicates subjects with total adiponectin level equal to or lower than a cut-off value and those with total adiponectin level higher than it. The optimal cut-off value of total adiponectin concentration between 3.1 and 8.3 μg/ml was investigated in 0.1 μg/ml increments. The cut-off value that gave the maximum log likelihood was determined as the optimal cut-off value. P<0.05 was considered statistically significant in all analyses. The SAS software package version 9.3 (SAS Institute, Cary, NC, USA) was used to perform all statistical analyses.

### Ethical considerations

This study was conducted with the approval of the Ethics Committee of Tokushima University Hospital, and written informed consent was obtained from the participants according to the Declaration of Helsinki.

## Results

Age-adjusted mean values or frequencies of MetS risk factors according to quartiles of total adiponectin level at baseline are summarized in Table 1. The median value of total adiponectin was 6.7 μg/ml in the total subjects. The mean values of BMI, waist circumference, and fasting plasma glucose and the geometric mean value of triglyceride decreased significantly with increasing total adiponectin level, whereas the mean value of HDL-C and the frequency of drinking habit increased significantly with increasing total adiponectin level.

**Table 1:** Age-adjusted mean values or frequencies of MetS risk factors according to quartiles of total adiponectin level at baseline.

	Total subjects	Total adiponectin level (μg/ml)				
		≤ 4.9	5.0 to 6.6	6.7 to 8.8	≥ 8.9	P for trend
	(n = 365)	(n = 90)	(n = 89)	(n = 94)	(n = 92)	
Age, years	40.4 ± 0.5	41.3 ± 0.9	39.3 ± 1.0	40.8 ± 0.9	40.0 ± 0.9	0.56
BMI, kg/m^2^	23.0 ± 0.1	24.0 ± 0.28	23.1 ± 0.28	23.0 ± 0.27	21.8 ± 0.28	<0.0001
Waist circumference, cm	80.9 ± 0.4	83.9 ± 0.78	81.5± 0.79	80.6 ± 0.77	77.6 ± 0.77	<0.0001
Fasting plasma glucose, mmol/l	4.96 (4.11–5.99)	5.06 (4.96–5.15)	4.96 (4.86–5.05)	4.95 (4.86–5.05)	4.88 (4.79–4.97)	0.01
HDL-cholesterol, mmol/l	1.50 ± 0.02	1.39 ± 0.03	1.42 ± 0.03	1.51 ± 0.03	1.69 ± 0.03	<0.0001
Triglycerides, mmol/l	1.01 (0.37–2.76)	1.26 (1.15–1.39)	1.10 (1.00–1.21)	0.98 (0.89–1.07)	0.79 (0.72–0.87)	<0.0001
Systolic blood pressure, mmHg	120 ± 0.7	119 ± 1.3	122 ± 1.3	121 ± 1.3	118 ± 1.3	0.45
Diastolic blood pressure, mmHg	79 ± 0.6	77 ± 1.2	81 ± 1.2	80 ± 1.2	77 ± 1.2	0.91
Hs-CRP, mg/l	0.45 (0.05–3.84)	0.61 (0.49–0.76)	0.44 (0.35–0.55)	0.47 (0.38–0.58)	0.32 (0.26–0.40)	0.0003
Smoking habit, %	25.8	19.8	27.5	29.4	24.9	0.43
Alcohol intake, %	57.5	46.4	55.9	59.3	67.9	0.005
Regular exercise, %	60.0	55.1	57.7	65.8	62.6	0.15

Values are presented as mean ± SE, geometric mean (95% CI) or percentage.

All values according to quartiles of total adiponectin are age-adjusted (except age).

MetS, metabolic syndrome; hs-CRP, high-sensitivity C-reactive protein

During a mean follow-up of 3.1 years, 45 men developed MetS. Time ratio (TR) and 95% CI for the development of MetS according to total adiponectin level at baseline based on the Weibull distribution are shown in Table 2. Age-adjusted TR significantly declined with decreasing total adiponectin level (≤ 4.9, 5.0–6.6, 6.7–8.8 and ≥ 8.9 μg/ml, P for trend = 0.003). In multivariate analysis, this association remained substantially unchanged even after adjustment for BMI, smoking habit, alcohol intake and regular exercise. Multivariate-adjusted TR of MetS was 0.12 (95% CI 0.02–0.78; P = 0.03) in subjects with total adiponectin level of 5.0–6.6 μg/ml, and 0.15 (95% CI 0.02–0.97; P = 0.047) in subjects with total adiponectin level ≤ 4.9 μg/ml compared with that in subjects with total adiponectin level ≥ 8.9 μg/ml.

**Table 2:** TR and 95% CI for development of MetS according to total adiponectin level at baseline based on Weibull distribution.

	Total adiponectin level (μg/ml)				P for trend
	≤ 4.9	5.0 to 6.6	6.7 to 8.8	≥ 8.9	(across category)
Median total adiponectin (μg/ml)	4.10	5.90	7.30	10.65	
No. of events/ population at risk	18/90	15/89	9/94	3/92	
Mean follow-up (years)	3.02	3.01	3.13	3.34	
Age-adjusted TR (95% CI)	0.07 (0.01–0.51)	0.08 (0.01–0.58)	0.20 (0.03–1.42)	ref	0.003
Multivariate-adjusted TR (95% CI)	0.15 (0.02–0.97)	0.12 (0.02–0.78)	0.30 (0.05–1.95)	ref	0.03

Multivariate adjustment was performed for age, BMI, smoking habit, alcohol intake, and regular exercise.

MetS, metabolic syndrome; TR, time ratio

To explore the optimal cut-off value of total adiponectin level for managing the risk of developing MetS, Table 3 summarizes the top five log likelihoods in each survival distribution, arranged in descending order for exponential, Weibull, and gamma distributions. The cut-off value of total adiponectin of 6.2 μg/ml had the maximum log likelihood in each distribution. The Weibull distribution had the minimum AIC among the three distributions, so that it provided the best fit to the data.

**Table 3:** Cut-off values of total adiponectin level for managing risk of developing MetS according to top five log likelihoods in each survival distribution.

	Rank order	Cut-off value of total adiponectin (μg/ml)	Log likelihood*	AIC*
Weibull distribution	1	6.2	-161.71	339.428
	2	6.3	-162.50	341.007
	3	6.1	-162.57	341.146
	4	6.4	-163.11	342.226
	5	6.5	-163.34	342.675
Exponential distribution	1	6.2	-163.19	340.389
	2	6.3	-163.99	341.986
	3	6.1	-164.11	342.210
	4	6.4	-164.61	343.223
	5	6.5	-164.80	343.598
Gamma distribution	1	6.2	-161.70	341.394
	2	6.3	-162.50	342.990
	3	6.1	-162.52	343.039
	4	6.4	-163.11	344.214
	5	6.5	-163.33	344.670

Cut-off values were confined to top five log likelihoods in each survival distribution.

* Comparison was made between subjects with total adiponectin level ≤ cut-off value and subjects with total adiponectin level > cut-off value.

Multivariate adjustment was performed for age, BMI, smoking habit, alcohol intake, and regular exercise.

MetS, metabolic syndrome; AIC, Akaike’s information criterion

We next estimated TR and 95% CI for the development of MetS in subjects with total adiponectin level ≤ 6.2 μg/ml compared with > 6.2 μg/ml based on the Weibull distribution (Table 4). With a cut-off value of total adiponectin of 6.2 μg/ml, the age-adjusted TR of MetS was 0.14 (95%CI: 0.04–0.46; P = 0.001). In multivariate analyses, after adjustment for age, BMI, smoking habit, alcohol intake and regular exercise, TR of MetS was 0.22 (95%CI: 0.08–0.64; P = 0.005). The number of events and the population at risk for each group were 33 events and 156 population at risk in subjects with total adiponectin level ≤ 6.2 μg/ml, and 12 events and 209 population at risk in subjects with total adiponectin level > 6.2 μg/ml. This association remained robust even after controlling for hs-CRP (TR 0.24: 95%CI 0.08–0.69, P = 0.008). Because TRs of MetS were estimated based on the Weibull distribution, they could be transformed to estimated hazard ratios [19]. Estimated hazard ratio (HR) was 3.99 in Model 1, 3.04 in Model 2, and 3.04 in Model 3.

**Table 4:** TR and 95% CI for development of MetS in subjects with total adiponectin level ≤ 6.2 μg/ml compared with > 6.2 μg/ml based on Weibull distribution.

	TR (95%CI)	P value	Estimated HR
No. of events/ population at risk	45/365		
Model 1	0.14 (0.04–0.46)	0.001	3.99
Model 2	0.22 (0.08–0.64)	0.005	3.04
Model 3	0.24 (0.08–0.69)	0.008	3.04

Model 1: adjusted for age. Model 2: model 1 plus additional adjustment for BMI, smoking habit, alcohol intake, and regular exercise. Model 3: model 2 plus additional adjustment for hs-CRP.

MetS, metabolic syndrome; TR, time ratio; HR, hazard ratio

## Discussion

In this prospective occupation-based study, we demonstrated that the mean time to the development of MetS declined with decreasing total adiponectin level. This study used an AFT model to analyze the mean time to the development of MetS. The onset of chronic diseases like MetS and diabetes could not be strictly identified. Thus, an AFT model including interval-censoring is more appropriate than a Cox proportional hazards model, which requires rigorous identification of the order of onset [11]. The optimal cut-off value of total adiponectin for managing the risk of developing MetS derived from the AFT model was 6.2 μg/ml. Moreover, the mean time to the development of MetS was 78% shorter in subjects with total adiponectin level ≤ 6.2 μg/ml compared with > 6.2 μg/ml. This association remained robust even after controlling for hs-CRP.

In this study, a significant inverse association between total adiponectin and MetS was observed in univariate and multivariate analyses. Similar inverse associations between MetS and adiponectin level have been reported mainly in cross-sectional studies [8, 9, 20], but data from prospective studies are sparse [21–23]. In addition, many studies included elderly persons, who are more likely to develop MetS. However, our findings, which were obtained from subjects whose mean age was approximately 40 years old, support the hypothesis that adiponectin has a preventive influence on MetS in young and middle-aged adults. Furthermore, the optimal cut-off value of total adiponectin is useful to manage the risk of developing MetS in clinical practice and preventive healthcare. The use of an optimal cut-off value would make it possible to predict the future development of MetS, which could enable initiation of early intervention even before any clinical data become abnormal. Although the precise reasons for this finding are incompletely understood, the effects of adiponectin on the development of MetS could be mediated via several possible mechanisms. Adiponectin is an adipocyte-derived secretory protein with molecular weight 30kDa that exists as a wide range of multimer complexes in the circulation [6]. Two major isoforms of its receptors have been identified. AdipoR1 is the predominant receptor in skeletal muscle and AdipoR2 is abundantly expressed in the liver [24]. Adiponectin stimulates activation of AMP activated protein kinase and peroxisome proliferator-activated receptor-α through these receptors, leading to increased insulin sensitivity, fatty acid combustion and energy consumption [25]. Adiponectin also promotes glucose homeostasis by maintaining functional beta cell mass [26]. Regarding lipid metabolism, adiponectin activates lipoprotein lipase and AMP activated protein kinase, which in turn increases synthesis of HDL-C and lipid uptake and decreases triglyceride accumulation, which may result in improved serum lipid profile [27]. Besides, adiponectin is associated with the inhibition of inflammation and oxidative stress [28]. Moreover, adiponectin stimulates activation of endothelial nitric-oxide synthase in the vascular endothelium, resulting in increased production of nitric oxide and modulated blood pressure [29]. A prior study in type 2 diabetes patients showed that decreased plasma adiponectin correlated with impaired insulin-stimulated nitric-oxide synthase activity and severity of insulin resistance [30]. Therefore, decreased adiponectin level may have an adverse effect on the development of MetS.

We observed a significant inverse association between adiponectin and MetS after adjustment for low-grade inflammation as measured by hs-CRP, which suggests that adiponectin is significantly associated with the development of MetS independent of low-grade inflammation, at least in part. Supportively, a Korean prospective study showed a similar association independent of low-grade inflammation [21]. It has been opined that adiponectin has a strong impact on a wide range of mechanisms of development of MetS, rather than only an impact on low-grade inflammation. The Korean prospective study also showed that adiponectin level had predictive ability for identification of subjects at risk of developing new-onset MetS, beyond that of information provided by the components of MetS. Thus, there can be no doubt that adiponectin has clinical importance as a useful biomarker of MetS in male Japanese workers.

Our findings suggest that the optimal cut-off value of total adiponectin for managing the risk of developing MetS is 6.2 μg/ml. Few previous studies have investigated the cut-off value of total adiponectin. One study in patients with coronary artery disease reported a total adiponectin level of less than 4.0 μg/ml as the cut-off value for hypoadiponectinemia [31]. Meanwhile, it was reported that the cut-off value to discriminate the existence of diabetes was 5.7 μg/ml in Chinese [32]. Moreover, the cut-off value of total adiponectin to discriminate the existence of MetS was reported to be 6.65 μg/ml in obese Japanese boys aged 8–13 years old [33]. Cut-off values to discriminate MetS or diabetes in the above cross-sectional studies were comparable to our findings, so that the optimal cut-off value in our results was considered to be a plausible value for managing the risk of developing MetS in Asians.

The strengths of the present study include its longitudinal population-based design and the use of an AFT model for evaluation. Some potential limitations of this study should be noted. First, this cohort study focused on business workers, who may differ in various ways from the general population. However, we recruited subjects with various job types and working practices, so it is likely that the findings on adiponectin are meaningful. In addition, the prevalence of MetS in our cohort was similar to the strongly suspected prevalence of MetS derived from the National Health and Nutrition Survey in Japan (2011), a representative study of the Japanese population (data not shown). Second, we analyzed total adiponectin in serum, while there are different molecular weight complexes. Although the HMW isoform was reported to be more relevant in the prediction of insulin resistance [10], measurements of the HMW isoform may not be suited to a large scale population-based study due to the need for an ELISA. Thus, we have demonstrated an association between serum total adiponectin level, which can be analyzed using automated laboratory test equipment, and the mean time to the development of MetS. Additionally Komura et al. reported that change in the HMW isoform reflected change in the total adiponectin level in patients with coronary artery disease [34]. Therefore, we believe that measurement of total adiponectin is equally useful to that of the HMW isoform. Third, the follow-up period was relatively short and the number of events was small. In such a situation, significant results could not be obtained without a strong relationship between adiponectin and the development of MetS. Hence our results support that adiponectin level might play an important role in managing the risk of developing MetS. Fourth, the sample size may not be large enough and the estimated cut-off value might be different when analyzed in a larger sample. Fifth, the age range is rather wide and a larger number of young to middle-aged subjects were included than in other studies. However, when stratified analysis by age groups was conducted (see S1 Table), the results in subjects aged 20–40 years and 40–60 years showed the same tendency (P for heterogeneity = 0.29). Therefore, it is important to control total adiponectin level in the management of MetS, regardless of age. Sixth, lifestyle habits have a potential impact on the plasma level of adiponectin [35, 36]. Although we have analyzed the stratified data by lifestyle habits, such as smoking and alcohol intake, we did not observe an influence of them on TR in this study (data not shown).

In conclusion, the present analysis has shown that the cut-off value for managing the risk of developing MetS is 6.2 μg/ml in male Japanese workers. The mean time to the development of MetS was 78% shorter in subjects with total adiponectin level ≤ 6.2 μg/ml compared with > 6.2 μg/ml; that is, subjects with total adiponectin level ≤ 6.2 μg/ml developed MetS more rapidly compared with those with total adiponectin level > 6.2 μg/ml. These findings provide a valuable insight into developing a strategy to prevent MetS. Intervention studies to examine this cut-off value are needed.

## Supporting Information

S1 Table TR and 95% CI for development of MetS in subjects with total adiponectin level ≤ 6.2 μg/ml compared with > 6.2 μg/ml based on Weibull distribution according to risk factors. Model 2: adjusted for age, BMI, smoking habit, alcohol intake, and regular exercise. MetS, metabolic syndrome; TR, time ratio; HR, hazard ratio.
(DOCX)
