# Supplementary material for: Cut-Off Value of Total Adiponectin for Managing Risk of Developing Metabolic Syndrome in Male Japanese Workers
Source: PLoS One. 2015 Feb 23;10(2):e0118373. doi: 10.1371/journal.pone.0118373 (PMC4337907; doi:10.1371/journal.pone.0118373)
Supplement: S1 Table — Model 2: adjusted for age, BMI, smoking habit, alcohol intake, and regular exercise. MetS, metabolic syndrome; TR, time ratio; HR, hazard ratio. (DOCX) [file pone.0118373.s001.docx]

S1 Table. TR and 95% CI for development of MetS in subjects with total adiponectin level ≤ 6.2 μg/ml compared with > 6.2 μg/ml based on Weibull distribution according to risk factors

|  | No. of events/ population at risk | TR (95%CI) | P for heterogeneity |
| --- | --- | --- | --- |
| **Age, 20-40 (years)** |  |  |  |
| Model 2 | 17/178 | 0.08 (0.006-1.04) |  |
| **Age, 40-60 (years)** |  |  |  |
| Model 2 | 28/187 | 0.36 (0.12-1.10) | 0.29 |

Model 2: adjusted for age, BMI, smoking habit, alcohol intake, and regular exercise.

MetS, metabolic syndrome; TR, time ratio; HR, hazard ratio
